# Association of Sodium‐Glucose Cotransporter 2 Inhibitors Treatment With Contrast‐Induced Acute Kidney Injury in Older Adults With T2D and CKD: A Propensity Matched Analysis

**DOI:** 10.1111/1753-0407.70243

**Published:** 2026-06-09

**Authors:** Jing Ma, Qing Yang, Xing Yang, Ke Liu, Xiaolei Chen, Fang Liu

**Affiliations:** ^1^ Department of Nephrology West China Hospital of Sichuan University Chengdu China; ^2^ Laboratory of Diabetic Kidney Disease, Department of Nephrology Kidney Research Institute, West China Hospital of Sichuan University Chengdu China

To the Editor,

1

Contrast‐induced acute kidney injury (CI‐AKI) is responsible for the third highest proportion of hospital‐acquired AKI and has been linked with increased risks of chronic kidney disease (CKD) progression, in‐hospital dialysis, and mortality [1, 2]. Older age, type 2 diabetes (T2D), and pre‐existing CKD markedly elevate the risk of CI‐AKI [3, 4, 5, 6]; however, targeted pharmacological preventive strategies remain limited [7]. Sodium‐glucose cotransporter 2 inhibitors (SGLT2‐Is) hold promise for preventing CI‐AKI [8, 9, 10], but their renoprotective effects in older, high‐risk adults with T2D and advanced CKD remain uncertain, as most previous trials enrolled patients with early‐stage CKD [11].

This retrospective analysis included adults receiving coronary angiography (CAG) or percutaneous coronary intervention (PCI) at West China Hospital of Sichuan University between February 2020 and February 2025. We identified 491 older adults (≥ 60 years) with T2D and CKD who had pre‐ and post‐procedure serum creatinine (Scr) measurements (Figure S1). The primary outcome was the in‐hospital incidence of CI‐AKI, defined by the European Society of Urogenital Radiology (ESUR) criteria (CI‐AKI_ESUR_), with the KDIGO Scr criteria (CI‐AKI_KDIGO_) used for sensitivity analysis [12, 13]. Participants were categorized as SGLT2‐Is users or nonusers based on their admission medication status. To balance baseline characteristics, SGLT2‐Is users were 1:1 matched to nonusers using propensity score matching (PSM).

The 491 participants had an average age of 75.5 years, 61% were male, and the mean estimated glomerular filtration rate (eGFR) was 41.9 mL/min/1.73 m^2^. The overall in‐hospital incidence of CI‐AKI was 18% (Table S1). After PSM, 111 SGLT2‐Is users were matched to 111 nonusers, and baseline characteristics were well‐balanced between the two groups. Among SGLT2‐Is users, 94.6% were treated with dapagliflozin and 5.4% with empagliflozin (Table S2). In the matched cohort, SGLT2‐Is users had a significantly lower risk of CI‐AKI. The incidence of CI‐AKI_ESUR_ was 9.9% in the SGLT2‐Is group compared with 26% in the nonusers' group (*p* = 0.003). The unadjusted odds ratio (OR) for CI‐AKI_ESUR_ was 0.33 (95% CI: 0.15–0.68; *p* = 0.004), and the adjusted OR was 0.31 (95% CI: 0.14–0.66; *p* = 0.003) after adjusting for age, sex, HbA1c, and high‐density lipoprotein (HDL). Notably, this risk reduction was more pronounced with dapagliflozin treatment (aOR: 0.26 for CI‐AKIESUR, 95% CI: 0.11–0.56, *p* = 0.001). This protective association was consistent when CI‐AKI was redefined using the KDIGO criteria (aOR: 0.41, 95% CI: 0.18–0.90; *p* = 0.03) (Figure 1).

**FIGURE 1:**
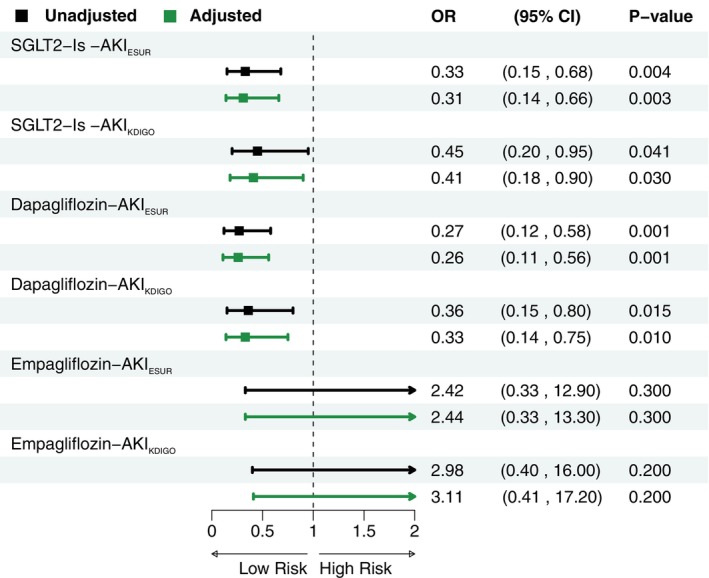
Association of CI‐AKI with SGLT2‐Is use: Unadjusted and adjusted analysis in the propensity score‐matched cohort. Covariates included in the adjusted model were age, gender, HbA1c and HDL. CI‐AKI, contrast induced acute kidney injury; HbA1c, glycated hemoglobin; HDL, high‐density lipoprotein; OR, odds ratios.

In secondary analyses, post‐procedural Scr was numerically higher in nonusers, though no significant intergroup difference was observed (Table S3). Multivariate analysis further identified baseline eGFR as an independent protective factor (Table S4). Subgroup analyses revealed that the protective effect was consistent across age groups and particularly marked in patients having an eGFR < 45 mL/min/1.73 m^2^ (OR = 0.33, 95% CI: 0.13–0.79, *p* = 0.015) (Figure S2).

Prior evidence indicates that SGLT2‐Is are well tolerated in older adults and may reduce AKI risk, yet concerns remain regarding their safety in those with substantially reduced eGFR [14, 15]. Our findings extend this evidence and suggest that SGLT2‐Is may serve as a prophylactic strategy against CI‐AKI in adults with T2D and CKD, especially in those with lower baseline renal function. These data provide clinical guideline for the use of SGLT2‐Is as a preventive strategy in patients with T2D and CKD undergoing CAG or PCI. This study is limited by its retrospective design, single‐center, and residual confounding; further prospective validation is warranted.

## Author Contributions

J.M., X.Y., and K.L. contributed to data collection. J.M. analyzed data. J.M. and Q.Y. drafted the manuscript. F.L. and X.C. were responsible for supervision. F.L. designed the study and were responsible for the manuscript review and editing. All authors read and approved the final manuscript.

## Funding

This study was supported by the Natural Science Foundation of Sichuan Province (Grant No. 2025ZNSFSC0006).

## Disclosure

The authors have nothing to report.

## Ethics Statement

The Ethics Committee of West China Hospital of Sichuan University approved this study (approval no. 202533), and informed or written consent was waived.

## Consent

The authors have nothing to report.

## Conflicts of Interest

The authors declare no conflicts of interest.

## Supporting information


**Figure S1:** Study population flowchart: unmatched and propensity score‐matched adults selection.
**Figure S2:** Subgroup analysis of the association between SGLT2‐Is use and CI‐AKI risk in the propensity score‐matched cohort.
**Table S1:** Baseline characteristics of the study population before and after propensity score matching.
**Table S3:** Comparison of the basic and post‐intervention characteristics of patients across two groups.
**Table S4:** Factors associated with CI‐AKI: univariate and multivariable logistic regression analysis.

## Data Availability

The data are not publicly available due to privacy and ethical restrictions.
