# Supplementary material for: Association of Sodium‐Glucose Cotransporter 2 Inhibitors Treatment With Contrast‐Induced Acute Kidney Injury in Older Adults With T2D and CKD: A Propensity Matched Analysis
Source: J Diabetes. 2026 Jun 9;18(6):e70243. doi: 10.1111/1753-0407.70243 (PMC13250466; doi:10.1111/1753-0407.70243)
Supplement: Supplementary file 1 — Figure S1: Study population flowchart: unmatched and propensity score‐matched adults selection. Figure S2: Subgroup analysis of the association between SGLT2‐Is use and CI‐AKI risk in the propensity score‐matched cohort. Table S1: Baseline characteristics of the study population before and after propensity score matching. Table S3: Comparison of the basic and post‐intervention characteristics of patients across two groups. Table S4: Factors associated with CI‐AKI: univariate and multivariable logistic regression analysis. [file JDB-18-e70243-s001.docx]

Supplementary Information

[Supplementary figure 1. Study population flowchart: unmatched and propensity score-matched adults selection. 2](#_Toc2112136158)

[Supplementary figure 2. Subgroup analysis of the association between SGLT2-Is use and CI-AKI risk in the propensity score-matched cohort. 2](#_Toc767722596)

[Supplementary Table 1. Baseline characteristics of the study population before and after propensity score matching. 3](#_Toc1031919796)

[Supplement Table 2.Basic characteritics of patients in SGLT2-Is users and non-users groups before and after propensity mactching. 4](#_Toc398078200)

[Supplementary Table 3. Comparison of the basic and post-intervention characteristics of patients across two groups. 6](#_Toc1088746995)

[Supplementary Table 4.Factors associated with CI-AKI: univariate and multivariable logistic regression analysis. 6](#_Toc2010072525)

#

# Supplementary figure 1. Study population flowchart: unmatched and propensity score-matched adults selection.


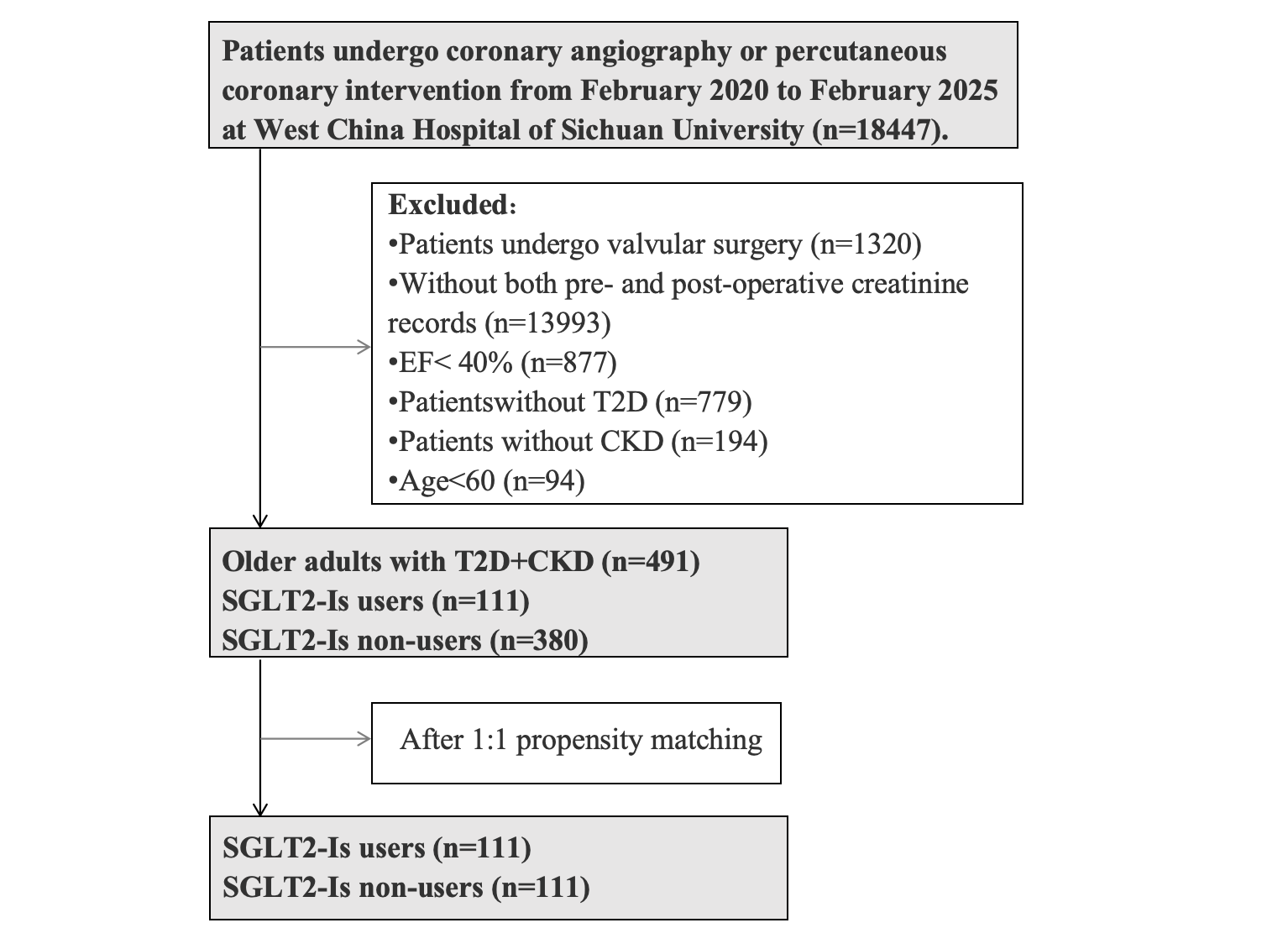


T2D, type 2 diabetes; CKD, chronic kidney disease; SGLT2-Is, sodium-glucose co-transporter 2 inhibitors.

# Supplementary figure 2. Subgroup analysis of the association between SGLT2-Is use and CI-AKI risk in the propensity score-matched cohort.


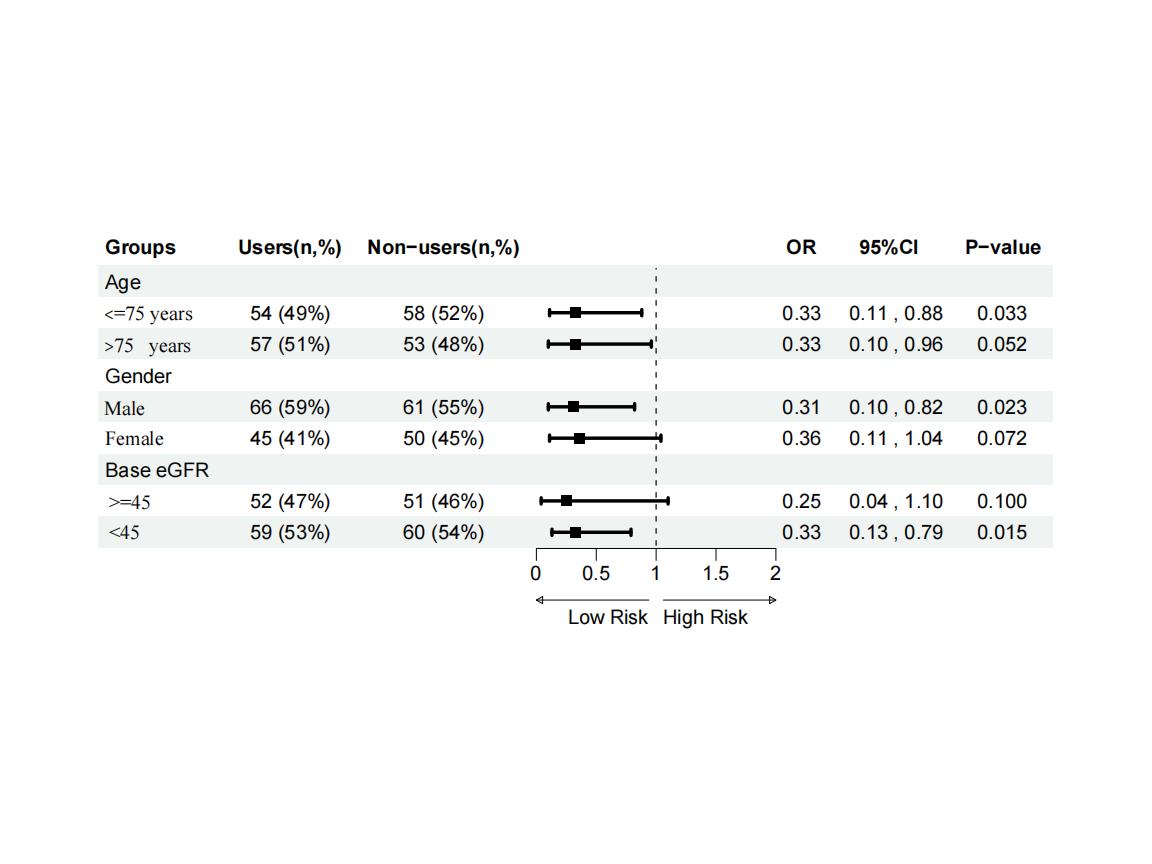


SGLT2-Is, sodium-glucose cotransporter 2 inhibitors; CI-AKI, contrast-induced acute kidney injury.

# Supplementary Table 1. Baseline characteristics of the study population before and after propensity score matching.

| Variables | Before matching （n=491） | After matching  (n = 222) |
| --- | --- | --- |
| **Demographics** | | |
| Age ,years | 75.5 ± 7.5 | 74.8 ± 7.5 |
| Male Sex | 300 (61%) | 127 (57%) |
| **Physiologic variables** | | |
| BMI, kg/m2 | 23.6 ± 3.4 | 24.1 ± 3.6 |
| SBP, mmHg | 136.1 ± 23.1 | 130.6 ± 23.5 |
| DBP, mmHg | 77.1 ± 13.3 | 73.9 ± 12.5 |
| **Comorbidities** | | |
| Smoking | 57 (12%) | 20 (9.0%) |
| Drinking | 29 (5.9%) | 12 (5.4%) |
| Hypertension | 453 (92%) | 206 (93%) |
| Dyslipidemia | 459 (93%) | 211 (95%) |
| Previous PCI | 91 (19%) | 46 (21%) |
| Coronary Artery Disease | 389 (79%) | 179 (81%) |
| COPD | 37 (7.5%) | 16 (7.2%) |
| **Medications** | | |
| RAAS | 118 (24%) | 58 (26%) |
| CCB | 192 (39%) | 76 (34%) |
| Β-blockers | 312 (64%) | 134 (60%) |
| Diuretics | 230 (47%) | 135 (61%) |
| Statins | 419 (85%) | 189 (85%) |
| Insulin | 436 (89%) | 194 (87%) |
| Metformin | 64 (13%) | 58 (26%) |
| Sulfonylureas | 13 (2.6%) | 11 (5.0%) |
| DPP4-Is | 21 (4.3%) | 14 (6.3%) |
| GLP-1 RAs | 14 (2.9%) | 11 (5.0%) |
| **Laboratory variables** | | |
| HbA1c,% | 6.9 (1.8) | 7.4 (2.0) |
| TC,mmol/L | 3.71 (1.46) | 3.76 (1.51) |
| TG,mmol/L | 1.53 (1.05) | 1.65 (1.33) |
| LDLmmol/L | 1.96 (1.09) | 2.01 (1.20) |
| HDL,mmol/L | 1.29 (1.21) | 1.32 (1.42) |
| UA,mg/dL | 404.7 ± 116.0 | 402.4 ± 123.2 |
| eGFR,ml/min/1.73㎡ | 41.9 (24.1) | 44.1 (20.8) |
| Scr,umol/L | 129(68.00) | 120(46.00) |
| Contrast dose,ml | 200 (200) | 200 (200) |
| Mehran score | 13 (6) | 12 (6) |
| **CI-AKI rate** | | |
| CI-AKIESUR | 87 (18%) | 39 (18%) |
| CI-AKIKDIGO | 80 (16%) | 33 (15%) |

Continuous variables are presented as mean ± SD or median (IQR), categorical variables are presented as number and percentage. BMI,Body mass index; SBP, Systolic blood pressure; DBP, Diastolic blood pressure; PCI, Percutaneous coronary intervention; COPD, Chronic obstructive pulmonary disease; RAASI, Renin-angiotensin-aldosterone system inhibitor; CCB, Calcium channel blocker; HbA1c, Glycated hemoglobin; TC, Total cholesterol; TG, Triglyceride; LDL, Low-density lipoprotein; HDL, High-density lipoprotein; UA, Uric acid; eGFR, Estimated glomerular filtration rate; Scr, Serum creatinine; EF, Ejection fraction; DPP4-Is, Dipeptidyl peptidase 4 inhibitors; GLP-1 RAs, Glucagon-like peptide-1 receptor agonists; CI-AKI, Contrast-induced acute kidney injury.

# Supplement Table 2.Basic characteritics of patients in SGLT2-Is users and non-users groups before and after propensity mactching.

| Variables | Before PSM | | |  | After PSM | | |
| --- | --- | --- | --- | --- | --- | --- | --- |
|  | Users  (n = 111) | Non-users  (n = 380) | p-Value |  | Users  (n = 111) | Non-users  (n =111) | p-Value |
| **Demographics** | | | | |  | | |
| Age ,years | 74.8 ± 7.6 | 75.7 ± 7.4 | 0.3 | | 74.8 ± 7.6 | 74.6 ± 7.1 | 0.8 |
| Male Sex | 66 (59%) | 234 (62%) | 0.8 | | 66 (59%) | 61 (55%) | >0.9 |
| **Physiologic variables** | | | | | | | |
| BMI, kg/m2 | 23.9 ± 3.3 | 23.5 ± 3.4 | 0.3 | | 24.1 ± 3.5 | 24.4 ± 3.5 | 0.5 |
| SBP, mmHg | 130.3 ± 23.1 | 137.8 ± 22.8 | **0.003** | | 130.7 ± 23.2 | 130.5 ± 23.6 | >0.9 |
| DBP, mmHg | 73.6 ± 12.2 | 78.1 ± 13.5 | **0.001** | | 73.7 ± 12.2 | 74.2 ± 11.4 | 0.8 |
| **Comorbidities** | | | | | | | |
| Smoking | 10 (9.0%) | 47 (12%) | 0.4 | | 10 (9.0%) | 7 (6.3%) | 0.6 |
| Drinking | 6 (5.4%) | 23 (6.1%) | >0.9 | | 6 (5.4%) | 6 (5.4%) | >0.9 |
| Hypertension | 104 (94%) | 349 (92%) | 0.7 | | 104 (94%) | 102 (92%) | 0.8 |
| Dyslipidemia | 107 (96%) | 352 (93%) | 0.2 | | 107 (96%) | 104 (94%) | 0.5 |
| Previous PCI | 26 (23%) | 65 (17%) | 0.2 | | 26 (23%) | 22 (20%) | 0.6 |
| Coronary Artery Disease | 90 (81%) | 299 (79%) | 0.7 | | 90 (81%) | 96 (86%) | 0.4 |
| COPD | 9 (8.1%) | 28 (7.4%) | >0.9 | | 9 (8.1%) | 6 (5.4%) | 0.6 |
| **Medications** | | | | | | | |
| RAAS | 25 (23%) | 93 (24%) | 0.8 | | 25 (23%) | 34 (31%) | 0.2 |
| CCB | 39 (35%) | 153 (40%) | 0.4 | | 39 (35%) | 37 (33%) | 0.9 |
| βblockers | 69 (62%) | 243 (64%) | 0.8 | | 69 (62%) | 67 (60%) | 0.9 |
| Diuretics | 68 (61%) | 162 (43%) | **<0.001** | | 68 (61%) | 64 (58%) | 0.7 |
| Statins | 95 (86%) | 324 (85%) | >0.9 | | 95 (86%) | 92 (83%) | 0.7 |
| Insulin | 94 (85%) | 342 (90%) | 0.2 | | 94 (85%) | 99 (89%) | 0.4 |
| Metformin | 34 (31%) | 30 (7.9%) | **<0.001** | | 34 (31%) | 24 (22%) | 0.2 |
| Sulfonylureas | 7 (6.3%) | 6 (1.6%) | **0.013** | | 7 (6.3%) | 4 (3.6%) | 0.5 |
| DPP4-Is | 8 (7.2%) | 13 (3.4%) | 0.11 | | 8 (7.2%) | 7 (6.3%) | >0.9 |
| GLP-1 RAs | 8 (7.2%) | 6 (1.6%) | **0.005** | | 8 (7.2%) | 5 (4.5%) | 0.6 |
| **Laboratory variables** | | | | | | | |
| HbA1c,% | 7.5 (2) | 6.7 (1.6) | **<0.001** | | 7.5 (2.0) | 7.3 (1.9) | 0.3 |
| TC,mmol/L | 3.75 (1.4) | 3.67 (1.5) | 0.2 | | 3.75 (1.39) | 3.83 (1.58) | >0.9 |
| TG,mmol/L | 1.60 (1.2) | 1.52 (1.0) | 0.12 | | 1.60 (1.22) | 1.61 (1.22) | 0.8 |
| LDL,mmol/L | 2.01 (1.1) | 1.95 (1.1) | 0.12 | | 2.01 (1.06) | 2.12 (1.28) | >0.9 |
| HDL,mmol/L | 1.32 (1.4) | 1.29 (1.1) | **0.029** | | 1.32 (1.41) | 1.24 (1.1) | **0.049** |
| UA,mg/dL | 390.5 ± 110.4 | 408.9 ± 117.4 | 0.13 | | 390.5 ± 110.4 | 403.1 ± 108.2 | 0.4 |
| eGFR,ml/min/1.73㎡ | 44.2 (20.8) | 41.1 (26.9) | **0.014** | | 44.2 (20.8) | 44.6 (20.4) | 0.8 |
| Scr,umol/L | 122.00 (45.0) | 132.50 (78.0) | **0.014** | | 122.00 (45.0) | 116.00 (49.0) | 0.2 |
| Contrast dose,ml | 200 (200.0) | 200 (200.0) | >0.9 | | 59.0 (17.0) | 60.0 (18.0) | 0.9 |
| Mehran score | 13 (6.0) | 14 (5.0) | 0.2 | | 200 (200.0) | 200 (200.0) | >0.9 |
| **SGLT2 inhibitors type** | | | | | | | |
| Dapagliflozin | 105 (94.6%) | - | - | | 105 (94.6%) | - | - |
| Empagliflozin | 6 (5.4) | - | - | | 6 (5.4) | - | - |
| **CI-AKI rate** | | | | | | | |
| CI-AKI_ESUR_ | 11 (9.9%) | 76 (20%) | 0.016 | | 11 (9.9%) | 29 (26%) | 0.003 |
| CI-AKI_KDIGO_ | 11 (9.9%) | 69 (18%) | 0.041 | | 11 (9.9%) | 23 (21%) | 0.04 |

Continuous variables are presented as mean ± SD or median (IQR), categorical variables are presented as number and percentage. BMI,Body mass index; SBP, Systolic blood pressure; DBP, Diastolic blood pressure; PCI, Percutaneous coronary intervention; COPD, Chronic obstructive pulmonary disease; RAASI, Renin-angiotensin-aldosterone system inhibitor; CCB, Calcium channel blocker; HbA1c, Glycated hemoglobin; TC, Total cholesterol; TG, Triglyceride; LDL, Low-density lipoprotein; HDL, High-density lipoprotein; UA, Uric acid; eGFR, Estimated glomerular filtration rate; Scr, Serum creatinine; EF, Ejection fraction; DPP4-Is, Dipeptidyl peptidase 4 inhibitors; GLP-1 RAs, Glucagon-like peptide-1 receptor agonists; CI-AKI, Contrast-induced acute kidney injury.

# Supplementary Table 3. Comparison of the basic and post-intervention characteristics of patients across two groups.

|  |  | Users | p-Value | Non-users | p-value | p-value for changes |
| --- | --- | --- | --- | --- | --- | --- |
| Scr  umol/L | Before | 122 (43) | 0.64 | 116 (47.5) | <0.01 | 0.12 |
|  | After | 124 (49.5) |  | 127 (59.5) |  |  |
|  | Changes | 1 (26) | - | 4 (32.5) | **-** |  |
| eGFRml/min/1.73㎡ | Before | 44.2 (20.3) | <0.01 | 44.5 (20.2) | <0.01 | 0.65 |
|  | After | 47.5 (23.9) |  | 48.5 (20.3) |  |  |
|  | Changes | 2.6 (11.1) | - | 2.2 (13.7) | - |  |

eGFR, Estimated glomerular filtration rate; Scr, Serum creatinine.

# Supplementary Table 4.Factors associated with CI-AKI: univariate and multivariable logistic regression analysis.

| Characteristics | Univariable | | |  | Multivariable | | |
| --- | --- | --- | --- | --- | --- | --- | --- |
|  | OR | 95CI% | p-value |  | OR | 95CI% | p-value |
| Age | 0.97 | 0.94, 1.01 | 0.12 |  | - | - | - |
| Male | 0.99 | 0.62, 1.60 | >0.9 |  | - | - | - |
| PCI | 1.18 | 0.65, 2.07 | 0.6 |  | - | - | - |
| eGFR | 0.97 | 0.95, 0.98 | **<0.001** |  | 0.97 | 0.96, 0.99 | **<0.001** |
| HbA1c | 1.15 | 1.00, 1.32 | **0.05** |  | 1.27 | 1.01, 1.48 | **0.002** |
| Insulin | 4.14 | 1.48, 17.3 | **0.019** |  | 3.83 | 1.14, 12.92 | **0.030** |
| Metformin | 0.36 | 0.12, 0.84 | **0.032** |  | 0.50 | 0.18, 1.39 | 0.182 |
| Mehran score | 1.03 | 0.98, 1.09 | 0.3 |  | - | - | - |
| SGLT2-Is | 0.44 | 0.21, 0.83 | 0.017 |  | 0.47 | 0.23, 0.98 | **0.044** |

PCI, precutaneous coronary intervention; eGFR, estimated glomerular filtration rate; HbA1c, glycated hemoglobin; SGLT2-Is, sodium-glucose cotransporter 2 inhibitors.
